# Attachment-Retained versus Clasp-Retained Removable Partial Dentures: Effects of Retention on Patient Satisfaction

**DOI:** 10.1055/s-0044-1795122

**Published:** 2024-12-30

**Authors:** Linda J. Dula, Tringa Z. Kelmendi, Kujtim Shala, Gloria Staka, Teuta Pustina- Krasniqi, Shera Kosumi

**Affiliations:** 1 Department of Prosthetic Dentistry, Faculty of Medicine, University of Prishtina, Prishtina, Republic of Kosovo; 2 Department of Dental Pathology and Endodontics, Faculty of Medicine, University of Prishtina, Prishtina, Republic of Kosovo; 3 Dental Faculty, Alma Mater Europaea Campus College “Rezonanca,” Prishtina, Republic of Kosovo

**Keywords:** removable partial dentures, retention, patient satisfaction, attachment-retained, clasp-retained, correlation

## Abstract

**Objectives** To compare the retention and patient satisfaction of attachment-retained versus clasp-retained removable partial dentures (RPDs) over time and to evaluate the impact of retention force on patient satisfaction.

**Materials and Methods** This study included 107 patients with 130 RPDs at the University Dentistry Clinical Center, Prishtina, Kosovo. Patients were divided into two groups: clasp-retained RPDs ( *n* = 79) and attachment-retained RPDs ( *n* = 51). RPD retention forces were measured using a dynamometer, and satisfaction was evaluated using a questionnaire covering retention, stability, chewing ability, aesthetics, oral hygiene maintenance, speech, and pain/discomfort on a Likert scale from 1 (complete dissatisfaction) to 5 (complete satisfaction). Reliability was assessed using Cronbach's α. Descriptive statistics and the independent-samples Kruskal–Wallis test were used for analysis, with pairwise comparisons and Spearman's rho correlation for additional insights.

**Results** Attachment-retained RPDs demonstrated superior retention, with mean scores decreasing from 5.43 to 4.40 over 3 months, compared with clasp-retained RPDs, which decreased from 4.02 to 3.23. Satisfaction scores also favored attachment-retained RPDs, dropping from 4.96 to 3.96, while clasp-retained RPDs decreased from 4.05 to 3.44. Cronbach's α indicated high reliability (α = 0.952). The Kruskal–Wallis test showed significant differences in retention and satisfaction between the two RPD types ( *p* < 0.0001). Pairwise comparisons indicated significant declines over time for both types. Spearman's rho correlation analysis revealed strong positive relationships between retention force and satisfaction scores, with correlation coefficients of 0.574 for clasp-retained and 0.522 for attachment-retained RPDs ( *p* < 0.0001).

**Conclusion** Attachment-retained RPDs offer higher and more stable retention and greater patient satisfaction compared with clasp-retained RPDs over the initial months of use. The significant positive correlation between retention force and patient satisfaction underscores the importance of optimizing retention in RPD design.

## Introduction

For partially edentulous patients missing posterior teeth, prosthetic options include fixed partial dentures, removable partial dentures (RPDs), and implant-supported dentures. 1 The key objective in prosthetic treatment is to preserve remaining teeth and supporting structures. 2 Although RPDs are a common solution for missing teeth, their retention can often be weak, leading to patient dissatisfaction. 3 McCracken's principles emphasize distributing forces to supporting tissues to ensure RPD stability and retention. 4

The retainer type is crucial for RPD retention, particularly in short dental arches where RPD arms transmit force to the remaining teeth. 5 Direct retainers, indirect retainers, and major connectors are essential for enhancing RPD retention and stability during oral functions. 6 7 8

Retention of RPDs can be achieved through the use of clasps or attachments that prevent the dentures from dislodging from the supporting structures. Clasp-retained RPDs offer benefits such as shorter fabrication time and lower costs, but they may be less aesthetically pleasing, especially for patients with Kennedy Class IV conditions, where the anterior teeth are missing. 9 10 Clasp arms on RPDs should be designed to flex easily and return to their original shape while providing adequate retention. Furthermore, they should not exert excessive stress on the supporting teeth or become permanently distorted over time. 11 Attachments, while improving RPD retention and stability, can sometimes allow for the removal of unaesthetic vestibular bracing arms, especially on the front upper teeth. 12

Patients assess RPDs based on their personal satisfaction with the restoration method. Key factors influencing acceptance of RPDs include retention, chewing ability, and aesthetics; these should be considered in any assessment tool for patient satisfaction. 13 Satisfaction is influenced by the patient's personality, previous experiences with RPDs, attitudes toward them, and the design and fabrication method used. 14 15 Dissatisfaction can arise from potential damage to the remaining teeth, such as caries, periodontal disease, and stomatitis. 16 17 Numerous studies indicate that most patients across different populations are generally satisfied with their RPDs. 18 19

RPDs are a noninvasive, reversible, and cost-effective treatment option, particularly for patients who cannot receive implant treatment due to anatomical or financial constraints. 20 21 This clinical study aims to enhance RPD design guidelines by evaluating the impact of retention in clasp-retained and attachment-retained RPDs on patient satisfaction.

This study operates under the null hypothesis that there is no significant difference in patient satisfaction or retention between clasp-retained and attachment-retained RPDs at various time points post-insertion.

## Materials and Methods

The study received approval from the Ethical Committee of the Faculty of Medicine, under approval number 1551. Written informed consent was obtained from all participants.

This research included 107 patients fitted with a total of 130 RPDs at the Department of Prosthodontics, University Dentistry Clinical Center, Prishtina, Kosovo. The patient group consisted of 49 females (45.8%) aged 32 to 73 years and 58 males (54.2%) aged 38 to 80 years. Inclusion criteria were no prior experience with RPDs, no tooth extractions in the past 3 months, good oral hygiene and a healthy periodontium, remaining teeth with ≤1 mm mobility, no history of diabetes or temporomandibular joint disorders, Angle class I jaw relationships, sufficient inter-arch space for RPD placement, and completion of all necessary dental treatments and restorations prior to RPD fitting.

### Designing Removable Partial Dentures and Prosthetic Procedures

Patients were categorized into two groups based on the type of RPD retainer: clasp-retained RPDs featuring extra-coronal direct and indirect Aker clasps ( *n* = 79) and attachment-retained RPDs utilizing Bar–Dolder attachments ( *n* = 51). The choice between clasp-retained and attachment-retained RPDs was based on patient preferences and financial considerations. Patients who chose clasp-retained RPDs were often unwilling to undergo tooth preparation or could not afford the higher cost of attachment-retained RPDs. All participants who met the inclusion criteria and consented to participate were included in the study. Frameworks were cast using cobalt–chrome–molybdenum alloys (Co-Cr-Mo). RPDs were classified based on Steffel's (1962) dental support categories: linear, triangular, and quadrangular. 22

The missing teeth were replaced with RPDs, each secured with two Acers clasps on the distal abutments on each side, following standardized clinical procedures. Depending on the specific denture support required, clasps were also placed on indirect abutment teeth. For anterior teeth, Bonyhard clasps were used. The RPD fabrication process involved several key steps. Initially, the abutment teeth were prepared to accommodate the partial denture. Impressions were then taken to capture the precise structure of the patient's oral cavity. A metal try-in was conducted to ensure proper fit and make any necessary adjustments. Once the fit was confirmed, artificial teeth were set up, and a wax try-in was conducted to evaluate occlusion. Finally, the completed RPD was delivered to the patient. Patients were provided with instructions on proper denture care and advised to return after 24 hours for a follow-up appointment to address any potential issues and prevent irritation.

Patients scheduled to receive RPDs alongside fixed bridges underwent initial tooth preparation for the bridge. Metal–ceramic bridges were fabricated following standard protocols, with impressions taken using polyvinyl siloxane material for enhanced accuracy. Fixed bridges were designed with two extra-coronal BAR-attachments, based on the Dolder system, and fabricated in the laboratory. The system consists of two primary components: male part: attached to the proximal surface of the crown on the abutment tooth, and female part: incorporated into the chrome–cobalt framework of the partial denture. This approach was selected primarily for cost-efficiency, as the attachments are provided at no charge in our clinic. Following this, new RPDs were created in line with standardized clinical procedures. A metal try-in was conducted to ensure proper fit and make necessary adjustments, artificial teeth were set up, and a wax try-in was performed to assess aesthetics and occlusion. After patient approval, the dentures were processed using heat-cured acrylic resin. The final cementation of the fixed bridge and delivery of the RPDs occurred once the dentures were complete. Patients were asked to return after 24 hours for a follow-up visit to address any concerns. Additionally, patients received comprehensive instructions on proper denture use and hygiene practices.

RPD retention force was measured with a dynamometer (Correx, Haag-Streit, Bern, Switzerland) with a range of 1 to 10 N. For clasp-retained RPDs, the dynamometer was placed in the cast clasps via a floss sling and pulled in the axial direction. For attachment-retained RPDs, it was placed on the buccal arm. Measurements were taken on the day of insertion and at 1 and 3 months post-insertion, averaging values from both sides of each RPD.

### Patient Satisfaction with Removable Partial Dentures

A newly developed questionnaire was used to assess patient satisfaction with RPDs. The questionnaire was designed specifically for this study and covered seven key aspects related to RPD performance and patient comfort: retention, stability, chewing ability, aesthetics, ability to maintain oral hygiene, ability to speak, and pain/discomfort. Each aspect was evaluated using a 5-point Likert scale, ranging from 1 (complete dissatisfaction) to 5 (complete satisfaction). 23

The development of the questionnaire was based on a comprehensive review of existing literature and insights from experienced prosthodontists. It was designed to capture the most relevant factors affecting patient satisfaction with RPDs. The questionnaire underwent a content validation process, in which a panel of experts reviewed the items for relevance, clarity, and comprehensiveness. Following the expert review, minor adjustments were made to improve the questionnaire's clarity and to ensure it appropriately reflected the core aspects of RPD performance.

The reliability of the final questionnaire was assessed using Cronbach's α coefficient, which showed a high level of internal consistency, with a Cronbach's α value of 0.952 and a Cronbach's α based on standardized items of 0.958 across the eight items.

### Data Analysis

Descriptive statistics, including mean and standard deviation (SD), were calculated for retention forces and patient satisfaction scores across different groups and time points. The sample size calculation was based on the expected effect size (medium effect size, ƒ = 0.25), power of the test (80%), significance level (0.05), and four groups (for different support types) to detect significant differences using the Kruskal–Wallis test, using G*Power statistical software. According to this calculation, at least 126 RPDs would be needed.

The independent-samples Kruskal–Wallis test was employed to evaluate the significance of differences in retention values and satisfaction scores between the two types of RPDs and among different types of denture support. This nonparametric test was chosen due to the ordinal nature of the satisfaction scores and the potential nonnormal distribution of retention force measurements, making it more appropriate than parametric tests for this analysis.

Pairwise comparisons were conducted to explore retention values among denture supports and over various time points, as well as to examine declines in retention and satisfaction scores over time. Additionally, Spearman's rho correlation analysis was performed to assess the relationship between retention force and patient satisfaction scores.

## Results

RPDs with attachments consistently demonstrated superior retention compared with clasp-retained RPDs at all measured time points. The mean retention score for attachment-retained RPDs was notably higher, starting at 5.43 immediately after insertion and moderately declining to 4.40 by 3 months. In contrast, clasp-retained RPDs experienced a more significant drop in retention, from an initial score of 4.02 down to 3.23 at 3 months. The lower SD values observed for attachment-retained RPDs throughout the study period suggest a more consistent and reliable performance.

There was a noticeable decrease over time for both types of dentures. Satisfaction scores for attachment-retained RPDs decreased from 4.96 immediately post-insertion to 3.96 at 3 months. Clasp-retained RPDs also showed a decrease, with scores moving from 4.05 to 3.44 over the same period. Despite the overall decline, attachment-retained RPDs still maintained higher satisfaction scores at each time point compared with clasp-retained RPDs, indicating a clear preference for attachment-based systems.

Overall, the data indicate that attachment-retained RPDs offer both higher and more stable retention forces, coupled with greater patient satisfaction, compared with their clasp-retained counterparts over the initial months of use ( Table 1 ).

**Table 1:** Descriptive statistics for retention force and patient satisfaction score with removable partial dentures (RPDs) by type and time point of measurements

		Retention force			Satisfaction score	
Point and measurement	Clasp-retained RPDs ( *n* = 79)	Attachment-retained RPDs ( *n* = 51)	Clasp-retained RPDs ( *n* = 79)	Attachment-retained RPDs ( *n* = 51)
Immediately after insertion (mean ± SD)	4.02 ± 0.71	5.43 ± 0.60	4.05 ± 0.64	4.96 ± 0.20
One month after insertion (mean ± SD)	3.70 ± 0.68	5.04 ± 0.55	3.77 ± 0.62	4.84 ± 0.37
Three months after insertion (mean ± SD)	3.23 ± 0.63	4.40 ± 0.50	3.44 ± 0.62	3.96 ± 0.53

Abbreviations: RPD, removable partial denture; SD, standard deviation.

**Fig. 1:**
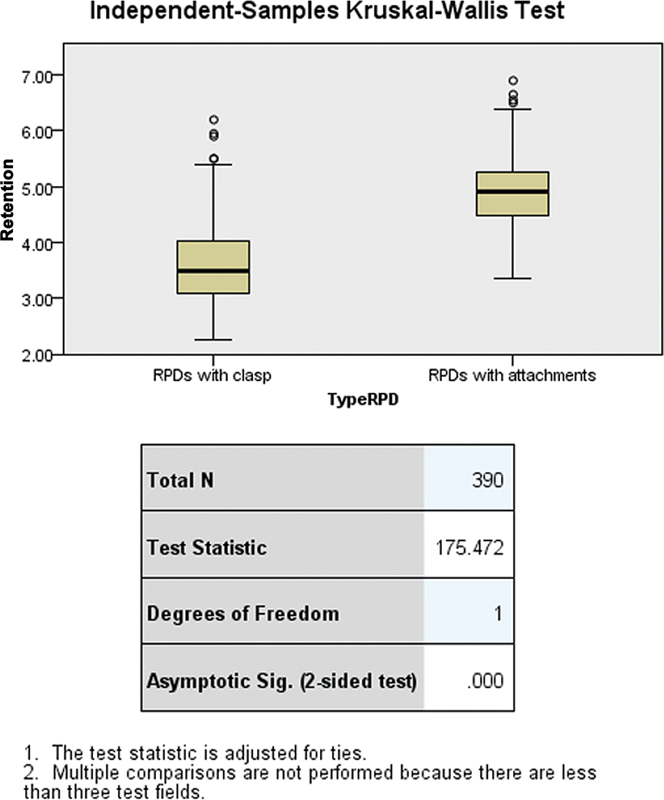
Statistical differences in retention value between clasp-retained and attachment-retained RPDs using the independent-samples Kruskal–Wallis test. RPD, removable partial denture.

This study employed the independent-samples Kruskal–Wallis test to assess the differences in retention values between clasp-retained and attachment-retained RPDs. The results, as illustrated in Fig. 1 , indicate statistically significant higher retention values for RPDs with attachments compared with those with clasps (test statistic = 175.472, df = 1, *p* < 0.0001).

**Fig. 2:**
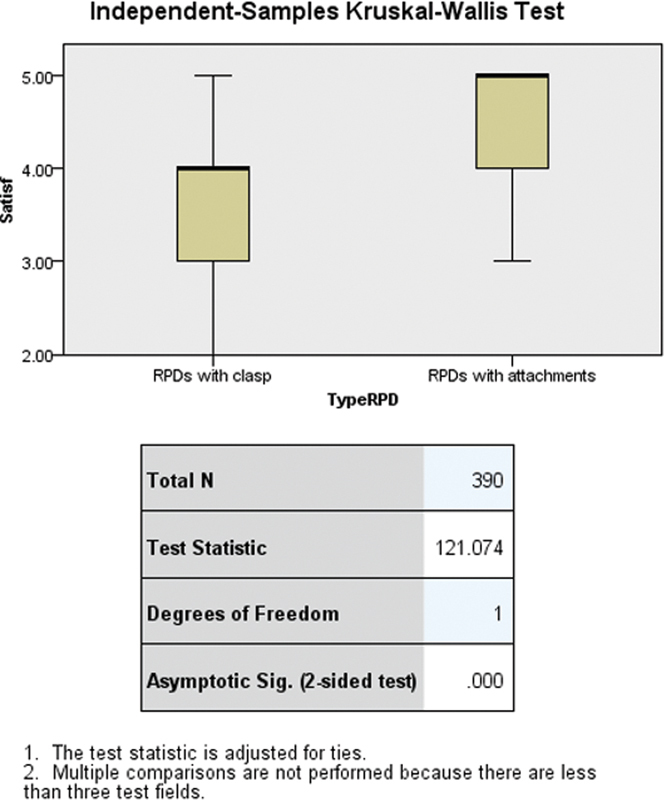
Statistical differences in satisfaction scores between clasp-retained and attachment-retained RPDs using the independent-samples Kruskal–Wallis test. RPD, removable partial denture.

Our study employed the Kruskal–Wallis test to evaluate differences in satisfaction levels between two types of RPDs: those with clasps and those with attachments. The results, as illustrated in the provided SPSS output ( Fig. 2 ), strongly suggest that the median satisfaction scores between clasp-retained and attachment-retained RPDs differ significantly (test statistic = 121.074, df = 1, *p* < 0.0001).

**Fig. 3:**
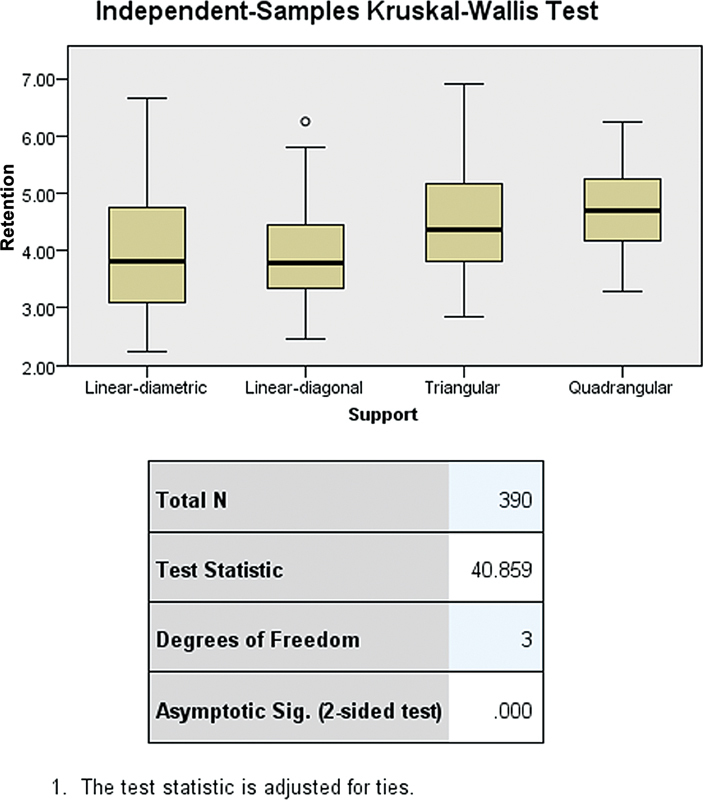
Statistical differences in retention value among different types of denture support in RPDs using the independent-samples Kruskal–Wallis test. RPD, removable partial denture.

The results of independent-samples Kruskal–Wallis test, depicted in ( Fig. 3 ), revealed a test statistic of 40.859 with a degree of freedom of 3, and an asymptotic significance ( *p* -value) of 0.000. This indicates statistically significant differences in retention values among the four RPD support types tested. The distribution of retention values, as illustrated in the box plots, shows variation in median retention and variability across the support designs, suggesting that the geometric configuration of the support significantly influences denture retention.

**Fig. 4:**
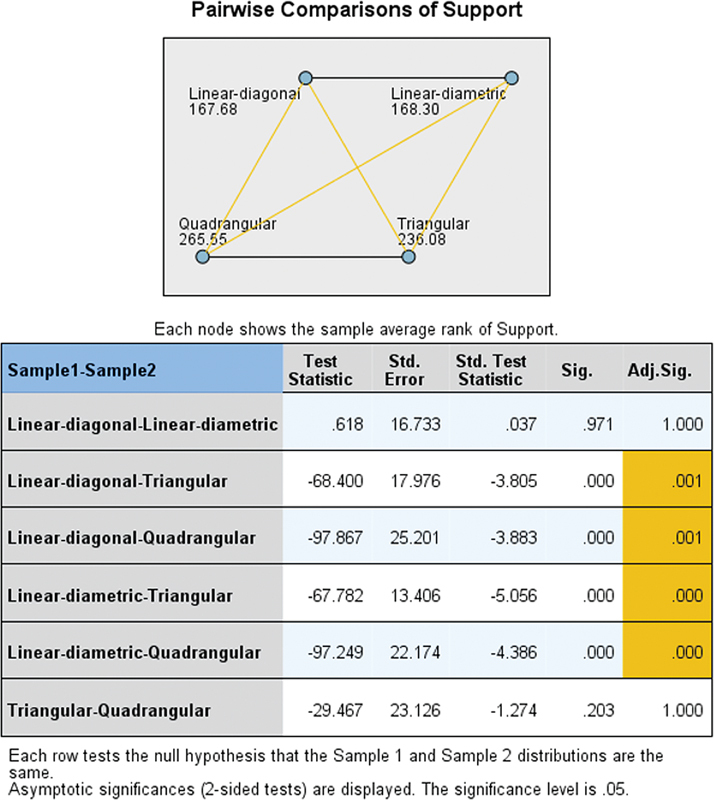
Pairwise comparisons of retention values among different types of denture supports in RPDs. RPD, removable partial denture.

The pairwise comparison chart and table indicate that the retention values significantly differ between linear-diagonal and triangular, linear-diagonal and quadrangular, linear-diametric and triangular, and linear-diametric and quadrangular supports, with no significant difference between linear-diagonal and linear-diametric, or between triangular and quadrangular supports in RPDs ( Fig. 4 ).

**Fig. 5:**
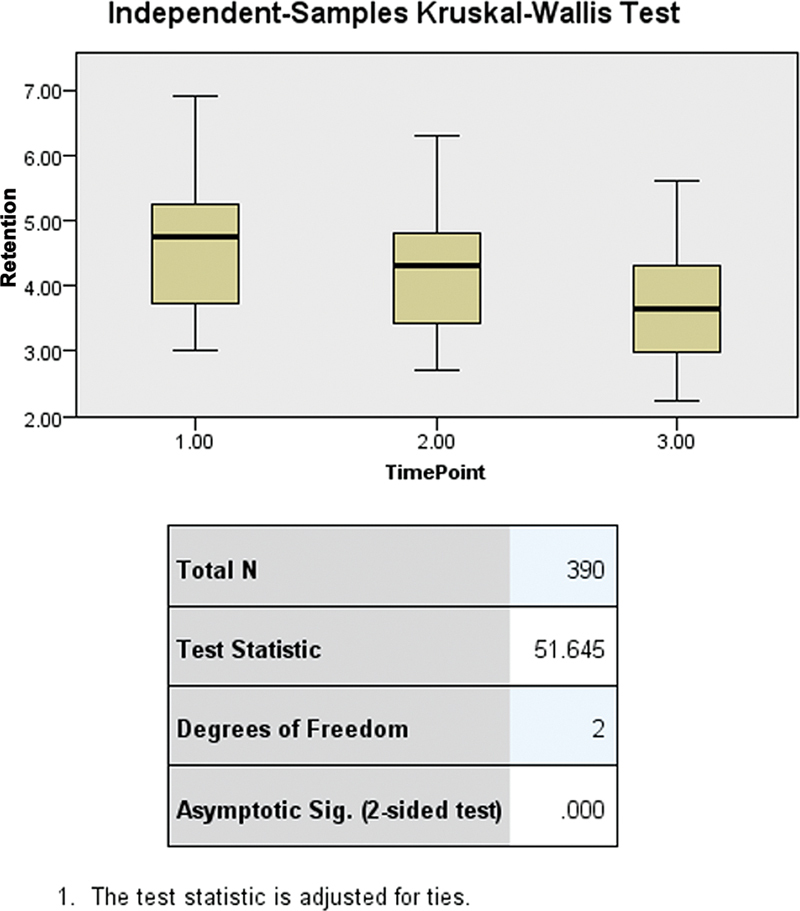
Statistical differences in retention values across various time points using the independent-samples Kruskal–Wallis test. RPD, removable partial denture.

The independent-samples Kruskal–Wallis test, with a test statistic of 51.645 and a *p* < 0.0001, confirms significant differences in retention values across three time points, indicating a decrease over time ( Fig. 5 ).

**Fig. 6:**
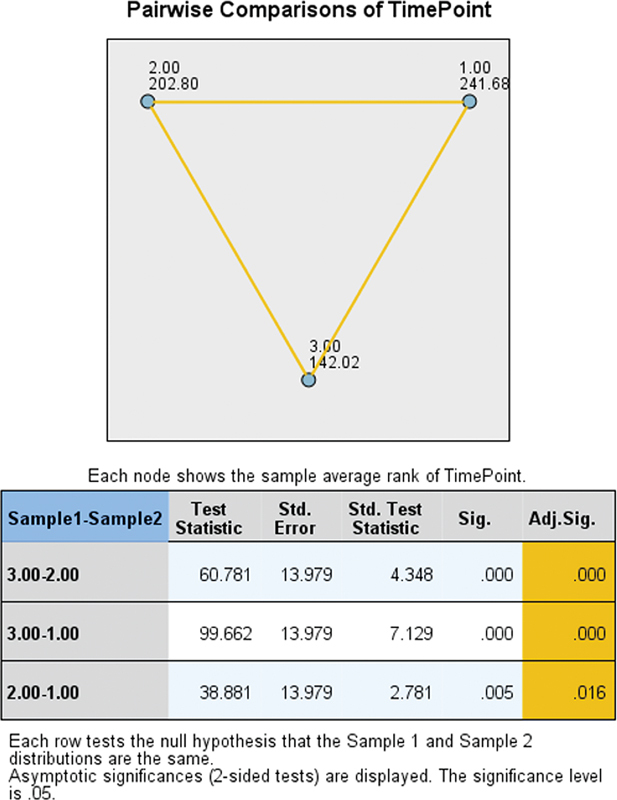
Pairwise comparisons of retention values among different time points of measurements in RPDs. RPD, removable partial denture.

The pairwise comparisons of retention values among different time points for RPDs demonstrate a statistically significant decrease over time, with the most pronounced decline observed from the initial to the final measurement period ( Fig. 6 ).

**Fig. 7:**
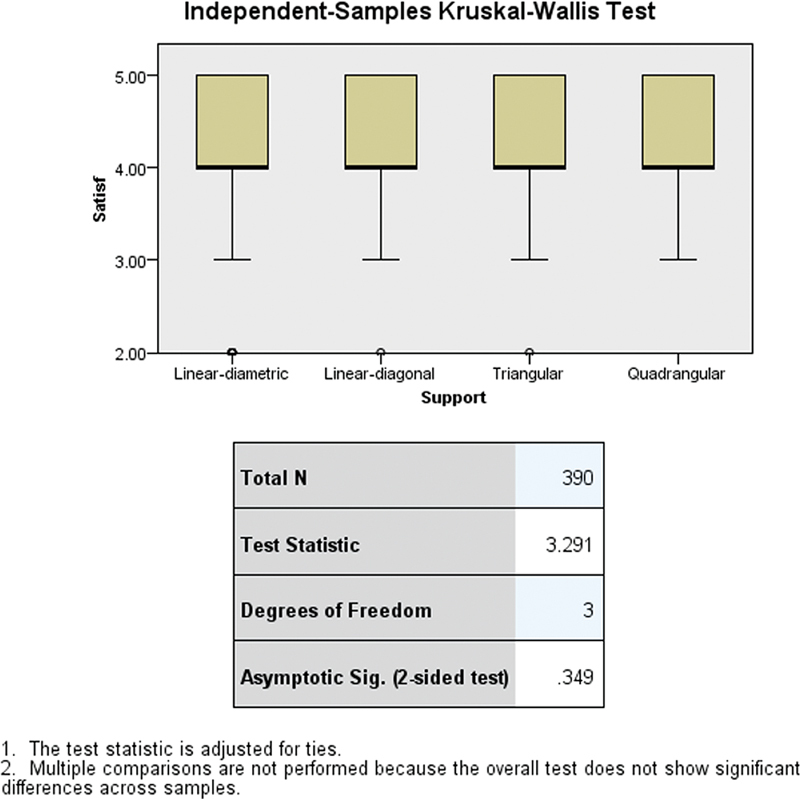
Statistical differences in satisfaction score among different types of denture support in RPDs using the independent-samples Kruskal–Wallis test. RPD, removable partial denture.

The results from the independent-samples Kruskal–Wallis test, as depicted in Fig. 7 , reveal no statistically significant differences in satisfaction scores among the different types of denture supports in RPDs. The test statistic of 3.291 with degrees of freedom being 3 results in an asymptotic significance (p-value) of 0.349, indicating that the variations in satisfaction scores across the linear-diametric, linear-diagonal, triangular, and quadrangular support types are not statistically significant.

**Fig. 8:**
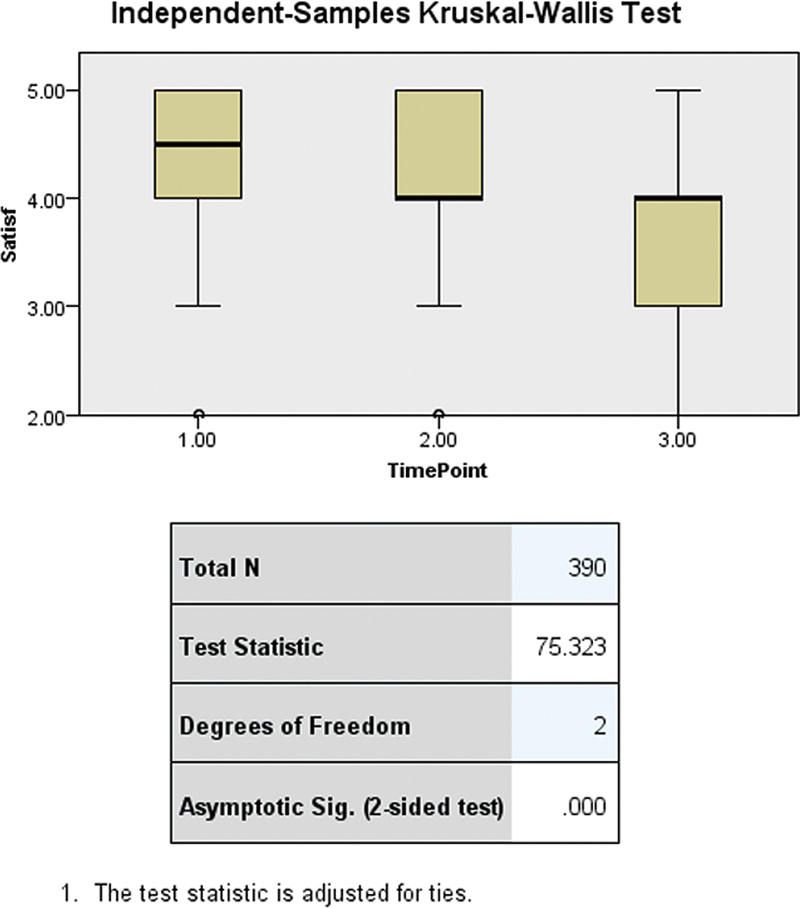
Pairwise comparisons of satisfaction score among different time points of measurements in RPDs. RPD, removable partial denture.

Pairwise comparisons revealed significant declines in satisfaction scores over time for RPDs, with the most pronounced reductions observed from the initial to the final time points (Time Point 1 to Time Point 3: test statistic = 108.704, *p* = 0.000). Decreases were also significant between intermediate and final time points (Time Point 2 to Time Point 3: test statistic = 77.642, *p* = 0.000) and from the first to the second time point (test statistic = 31.062, *p* = 0.016), underscoring a progressive decline in patient satisfaction with RPDs over the study period ( Fig. 8 ).

The results from Spearman's rho correlation analysis presented in Table 2 indicate statistically significant and strong positive correlations between retention force and patient satisfaction scores across various types of RPDs and denture support types. Specifically, RPDs with clasps showed a correlation coefficient of 0.574 ( *p* < 0.0001), and RPDs with attachments exhibited a correlation of 0.522 ( *p* < 0.0001), both indicating moderate to strong relationships. Among denture support types, linear-diametric supports displayed the strongest correlation at 0.812 ( *p* < 0.0001), followed by linear-diagonal supports at 0.641 ( *p* < 0.0001), triangular supports at 0.590 ( *p* < 0.0001), and quadrangular supports at 0.537 ( *p* = 0.002). These findings highlight the significant impact of both RPD type and support design on patient satisfaction, driven primarily by variations in retention force.

**Table 2:** Spearman's rho correlation coefficients between retention force and patient satisfaction score across different types of RPDs and denture support types

Type/support	Correlation coefficient	Significance (two-tailed)
RPDs with clasp	0.574 a	<0.0001
RPDs with attachments	0.522 a	<0.0001
Linear-diametric support	0.812 a	<0.0001
Linear-diagonal support	0.641 a	<0.0001
Triangular support	0.590 a	<0.0001
Quadrangular support	0.537 a	0.002

Abbreviation: RPD, removable partial denture.

a Indicates correlations significant at the 0.01 levels (two-tailed).

## Discussion

Numerous studies have highlighted the necessity of keeping RPD designs straightforward, 24 as complex designs can exert excessive force on supporting teeth, adversely affecting oral hygiene, patient comfort, and aesthetics. 25 In our study, we observed that attachment-retained RPDs provided better retention than clasp-retained RPDs, though both types showed a gradual decline in retention over time. The statistically significant differences in retention values between clasp-retained and attachment-retained RPDs, as demonstrated by the Kruskal–Wallis test (test statistic = 175.472, df = 1, *p* < 0.0001), underscore the critical role that RPD type plays in influencing both retention and patient satisfaction. The higher median retention values for attachment-retained RPDs indicate more consistent and stable performance over time compared with clasp-retained RPDs, confirming their advantages in clinical settings. These findings highlight the potential of attachment-retained dentures in cases where long-term retention and patient satisfaction are prioritized, offering valuable insights for dental practitioners and researchers aiming to optimize treatment outcomes ( Table 3 ).

**Table 3:** Descriptive statistics for retention force and satisfaction score by type of RPDs, type of supports, and time point of measurements

Support type	RPDs with clasp (mean ± SD)				RPDs with attachments (mean ± SD)			
	*n*	Immediately after insertion	One month after insertion	Three months after insertion	*n*	Immediately after insertion	One month after insertion	Three months after insertion
Retention								
Linear-diametric	34	3.54 ± 0.40	3.24 ± 0.37	2.82 ± 0.35	28	5.26 ± 0.50	4.89 ± 0.43	4.31 ± 0.35
Linear-diagonal	13	3.82 ± 0.37	3.53 ± 0.38	3.13 ± 0.42	7	5.16 ± 0.54	4.78 ± 0.55	4.19 ± 0.59
Triangular	26	4.53 ± 0.62	4.18 ± 0.58	3.63 ± 0.55	12	5.97 ± 0.58	5.51 ± 0.57	4.74 ± 0.62
Quadrangular	6	4.96 ± 0.74	4.66 ± 0.74	4.08 ± 0.80	4	5.51 ± 0.53	5.13 ± 0.46	4.36 ± 0.54
Satisfaction								
Linear-diametric	34	3.79 ± 0.59	3.56 ± 0.61	3.24 ± 0.55	28	4.96 ± 0.19	4.89 ± 0.32	4.00 ± 0.39
Linear-diagonal	13	4.15 ± 0.69	3.85 ± 0.69	3.46 ± 0.66	7	4.86 ± 0.38	4.71 ± 0.49	3.71 ± 0.49
Triangular	26	4.31 ± 0.62	3.96 ± 0.60	3.58 ± 0.64	12	5.00 ± 0.00	4.83 ± 0.39	4.08 ± 0.79
Quadrangular	6	4.17 ± 0.41	4.00 ± 0.00	4.00 ± 0.00	4	5.00 ± 0.00	4.75 ± 0.50	3.75 ± 0.50

Abbreviations: RPD, removable partial denture; SD, standard deviation.

Significant differences in retention values were found between the four RPD support designs—linear-diametric, linear-diagonal, triangular, and quadrangular—(test statistic = 40.859, df = 3, *p* < 0.0001). This indicates that the geometric configuration of the support plays an important role in optimizing denture retention and stability. Careful selection and customization of RPD support design can lead to improved retention outcomes, enhancing the functionality and stability of the dentures. These findings provide crucial guidance for clinical decision-making, enabling practitioners to tailor RPD designs to the individual needs of patients to maximize retention and satisfaction.

Among clasp-retained RPDs, those with triangular and quadrangular support exhibited the highest retention forces at all measured intervals, with significant variations across time points. However, at 3 months post-insertion, attachment-retained RPDs with triangular dental support had the highest retention, although this difference was not statistically significant. These results further emphasize the critical role that support design plays in denture retention and the need for careful customization to individual patients.

RPD structures made from metals and metal alloys, such as Co–Cr, experience continuous deformation and fatigue under stress. 26 Chewing forces vary depending on factors such as tooth type, occlusion degree, and food type. 27 It is documented that normal chewing can generate thousands of stress cycles daily. 28 The loss of retention in Co–Cr clasp-retained RPDs is often due to dental clasp fatigue during RPD insertion and removal, continuous clasp deformation on supporting teeth, patient misuse, and technician-induced mechanical adaptations. 29 30 Similarly, wear and tear on attachments result in decreased retention for attachment-retained RPDs over time. 31 Our findings align with these previous observations, noting a decrease in retention force with repeated use of RPDs. 32

Regarding patient satisfaction, our results indicated that patients with attachment-retained RPDs reported higher satisfaction scores than those with clasp-retained RPDs. This is consistent with previous studies (Alageel et al), who suggested that RPD retention determined by the number and placement of clasps, attachments, and missing teeth can predict patient satisfaction. 33 In our study, a significant positive correlation was found between retention rates and patient satisfaction scores at all three time points, although the strength of this correlation diminished over time. Attachment-retained RPDs maintained higher mean retention rates compared with clasp-retained RPDs. These findings support previous research, 34 which showed higher satisfaction rates among patients with attachment-retained RPDs (93.8%) compared with those with clasp-retained RPDs (58.7%).

Interestingly, no statistically significant differences in satisfaction scores were found across different support designs (test statistic = 3.291, df = 3, *p* = 0.349), suggesting that support type may not play a major role in patient satisfaction within this sample. This highlights that other factors, such as comfort, aesthetics, and fit, may be more critical in shaping patient experiences with RPDs. These findings underscore the complexity of patient satisfaction, which is influenced by a combination of clinical and subjective factors beyond the geometric configuration of the support design.

Our results are further supported by Peršić et al, 35 who found that satisfaction with aesthetics, comfort, stability during mastication, and speech was higher in patients with attachment-retained RPDs compared with those with clasp-retained RPDs. 35 36 However, the use of attachment-retained RPDs requires more technical and clinical expertise, leading to increased treatment costs. 37 Despite the advent of implant therapies, RPDs continue to be a favored treatment option for partially edentulous patients missing posterior teeth. 38 Retention elements vary based on treatment contexts, and more observational clinical studies and randomized clinical trials are needed to compare retention elements comprehensively. Due to their complexity and need for customization based on the type of edentulism, research on RPDs has been limited. 39

This study has several limitations. First, while the primary objective was to evaluate the impact of retention in clasp-retained and attachment-retained RPDs on patient satisfaction, we did not account for potential gender differences. Research has shown that female patients may report different levels of satisfaction, particularly regarding aesthetics and functional outcomes such as chewing ability. Therefore, future studies should include an analysis of gender-specific differences to gain a more comprehensive understanding of patient satisfaction with RPDs. Second, the status of the opposing dentition was not assessed in this study. The condition of the opposing jaw can significantly influence chewing ability, speech, and overall functionality, which are critical components of patient satisfaction. Future research should consider the impact of opposing dentition to provide more accurate insights into patient satisfaction outcomes related to RPDs.

Jacobson emphasized that RPD design guidelines should focus on minimal tooth coverage by the metal framework, elimination of unnecessary components, and maintenance of biomechanical principles. 40 An ideal RPD design should minimize stress on retained teeth and the alveolar ridge to ensure that normal occlusal forces do not harm the retained teeth, regardless of the retainer type. 41 Therefore, evaluating RPD outcomes is crucial for influencing patient satisfaction.

## Conclusion

Attachment-retained RPDs offer higher and more stable retention and greater patient satisfaction compared with clasp-retained RPDs over the initial months of use. The significant positive correlation between retention force and patient satisfaction underscores the importance of optimizing retention in RPD design.
